# Comparative assessment and QA measurement array validation of Monte Carlo and Collapsed Cone dose algorithms for small fields and clinical treatment plans

**DOI:** 10.1002/acm2.14522

**Published:** 2024-09-17

**Authors:** Guus B. Spenkelink, Sophie C. Huijskens, Jaap D. Zindler, Marc de Goede, Wilhelmus J. van der Star, Jaap van Egmond, Anna L. Petoukhova

**Affiliations:** ^1^ Haaglanden Medical Center, Department of Medical Physics Leidschendam The Netherlands; ^2^ Haaglanden Medical Center, Department of Radiation Oncology Leidschendam The Netherlands; ^3^ HollandPTC, Department of Radiotherapy Delft The Netherlands

**Keywords:** ArcCHECK, Collapsed Cone algorithm, Monte Carlo algorithm, small field dosimetry

## Abstract

**Purpose:**

Many studies have demonstrated superior performance of Monte Carlo (MC) over type B algorithms in heterogeneous structures. However, even in homogeneous media, MC dose simulations should outperform type B algorithms in situations of electronic disequilibrium, such as small and highly modulated fields.

Our study compares MC and Collapsed Cone (CC) dose algorithms in RayStation 12A. Under consideration are 6 MV and 6 MV flattening filter‐free (FFF) photon beams, relevant for VMAT plans such as head‐and‐neck and stereotactic lung treatments with heterogeneities, as well as plans for multiple brain metastases in one isocenter, involving highly modulated small fields. We aim to investigate collimator angle dependence of small fields and performance differences between different combinations of ArcCHECK configuration and dose algorithm.

**Methods:**

Several verification tests were performed, ranging from simple rectangular fields to highly modulated clinical plans. To evaluate and compare the performance of the models, the agreements between calculation and measurement are compared between MC and CC. Measurements include water tank measurements for test fields, ArcCHECK measurements for test fields and VMAT plans, and film dosimetry for small fields.

**Results and conclusions:**

In very small or narrow fields, our measurements reveal a strong dependency of dose output to collimator angle for VersaHD with Agility MLC, reproduced by both dose algorithms.

ArcCHECK results highlight a suboptimal agreement between measurements and MC calculations for simple rectangular fields when using inhomogeneous ArcCHECK images. Therefore, we advocate for the use of homogeneous phantom images, particularly for static fields, in ArcCHECK verification with MC.

MC might offer performance benefits for more modulated treatment fields. In ArcCHECK results for clinical plans, MC performed comparable to CC for 6 MV. For 6 MV FFF and the preferred homogeneous phantom image, MC resulted in consistently better results (13%–64% lower mean gamma index) compared to CC.

## 1 INTRODUCTION

The accuracy of dose calculations in radiation therapy (RT) treatment planning is of paramount importance in ensuring the desired target dose while minimizing dose to healthy tissues. Various dose calculation algorithms exist, each addressing specific challenges between interaction of radiation beams and biological tissues. These algorithms are generally classified as type A, B, and C, depending on their complexity in managing charged particle transport.^1^, ^2^ Type A and B encompass all pencil beam algorithms and Collapsed Cone (CC) convolution algorithms,^3^ respectively, while type C algorithms primarily include Monte Carlo algorithms (MC) for photons^4^ and the Linear Boltzmann Transport Equation solver.^5^ Among these, MC dose calculations present the highest level of accuracy in dose computation.^6^ Particularly, due to its ability to model lateral electron transport, even in the case of small fields with a loss of lateral charged particle equilibrium.^7^, ^8^ With growing use of hypofractionated and stereotactic radiotherapy using non‐coplanar volumetric‐modulated arc therapy (VMAT), combined with complex and small treatment fields, critical validation of beam modeling for these dose engines is essential.

The primary objective of this study is to compare verification measurements and calculations between two prominent dose engines implemented in RayStation v.12A (RaySearch Laboratories, Stockholm, Sweden): MC and CC, with a focus on small fields, collimator angle rotation dependence for small fields, and phantom validation.

AAPM Task Group 157 has outlined comprehensive guidelines for beam modeling and beam model commissioning for MC‐based treatment planning.^9^ A well‐known combination of commissioning for MC with a VersaHD accelerator (Elekta, Stockholm, Sweden) is in Monaco treatment planning system (Elekta, Stockholm, Sweden).^10^, ^11^ The commissioning and validation of RayStation CC convolution superposition and Elekta accelerators (Elekta, Stockholm, Sweden) equipped with Agility multi‐leaf collimators (MLCs) has been described in previous studies by Mzenda et al.^12^ and Manco et al.^13^ Photon MC models for Varian accelerators have been described by Richmond et al.^14^ for a Truebeam accelerator (Varian, Palo Alto, USA), and Saini et al.^15^ for a Halcyon accelerator (Varian, Palo Alto, USA) in earlier versions of RayStation. The implementation and validation of the MC dose algorithm in RayStation for Elekta VersaHD accelerator with Agility MLC, as detailed in this study, remains less explored, with only one study by Manco et al.^13^ to our knowledge, in an earlier version of RayStation.

While many studies have demonstrated that MC‐based dose algorithms are better suited to calculate dose distributions in heterogeneities as compared to CC algorithms,^13^, ^14^, ^16^, ^17^ our study specifically examines and compares their performance in mostly homogeneous phantoms [water tank and homogeneous ArcCHECK (Sun Nuclear Corporation, Melbourne, Florida, USA)], with a focus on small fields.

Petoukhova et al.^18^ have used ArcCHECK to compare the Monte Carlo and Pencil Beam dose algorithms in iPlan RT Dose (BrainLab, Munich, Germany) and found MC to be superior for intensity‐modulated arc therapy. Valdenaire et al.^19^ used ArcCHECK to measure VMAT plans calculated with CC in RayStation and MC of Monaco treatment planning system. But to our knowledge, such extensive study of MC calculations on ArcCHECK in RayStation has not been reported in the literature. Yani et al.^20^ have used ArcCHECK verification for 3D‐CRT plans calculated with MC code, but presented only a qualitative comparison of dose distributions. In contrast, our study provides an extensive quantitative analysis.

Furthermore, to our knowledge, the effect of collimator angle on small‐field dose output for Elekta VersaHD accelerators has not yet been studied and reported on in literature.

This study includes both 6 MV and 6 MV flattening filter free (FFF) photon beams, which are clinically used for complex VMAT treatment plans, such as stereotactic lung plans and head‐and‐neck plans whose treatment sites contain various heterogeneities, as well as treatment plans for multiple brain metastases, which are highly modulated plans with small fields.

Emphasizing the specific challenges posed by small fields, collimator angle dependence of small fields, film dosimetry, and ArcCHECK (Sun Nuclear, Melbourne, USA) verification measurements, the comparative analysis presented in this study seeks to provide new insights into the strengths and limitations of MC and CC algorithms.

## 2 MATERIALS AND METHODS

A number of verification tests were performed, with complexity ranging from simple rectangular fields to highly modulated clinical treatment plans. For each test, three results were obtained: a physical measurement performed on a linear accelerator (LINAC), a treatment planning system (TPS) calculation using CC, and a TPS calculation using MC. In order to evaluate and compare the performance of the models, the agreement between simulation and measurement is compared between the two dose algorithms.

### 2.1 Collapsed Cone and Monte Carlo calculations

Calculations were performed in RayStation 12A (RaySearch Laboratories, Stockholm, Sweden), which features both a CC (CC Dose, version 5.7) and a MC (MC Dose, version 2.0) dose algorithm.

In the CC dose algorithm in RayStation, the primary source is a spatially elliptic Gaussian, and the secondary source (flattening filter source) is a circular Gaussian. For contaminating electrons, there are also two sources. The first one has the same shape as the secondary photon source while the second one is a circular Gaussian source, which represents the electrons created in the air. The energy resulting from the attenuation of primary photons in each voxel, taking into account the material properties is further spread out using pre‐computed EGSnrc kernels combining all relevant physical processes in water.^21^, ^22^

The MC dose algorithm in RayStation applies the same dual virtual source head model as the CC model, without the use of any external packages. The MC code is a Class B algorithm (condensed history) and transports photons, electrons, and positrons. For photons, Compton scattering, photoelectric absorption, and pair creation events are simulated. The MC in‐patient transport is inspired by the EGSnrc code.^22^ The fictitious cross‐section method is used to avoid traversing each voxel (also known as Woodcock tracking).^23^

For both dose engines, dose models for 6 MV (nominal 6 MV) and 6 MV FFF (nominal 6 MV) photon beams of a VersaHD accelerator with Agility MLC were commissioned in‐house based on measurements in water (see for details of water tank measurements 2.2.1). In the TPS, beam models are commissioned by the user by adjusting a set of tuning parameters in order to optimize the model fit to water tank measurements. Parameters include the photon and electron energy spectra, physical parameters describing the source, flattening filter and collimator, and off‐axis corrections in beam softening and beam profile. In Raystation, this set of tunable parameters is identical for both dose algorithms.^24^ The parameter values of our clinical CC beam models were used as a starting point for tuning the MC beam models. Still, parameters had to be adjusted to get a comparably good fit in the MC model. For the 6 MV model, the following changes were made relative to the CC model: the energy spectrum was changed by increasing the fluence at 0.5 MeV by about 90%, the beam profile correction factors were decreased by 2%–3% starting from 10 cm off‐axis, and more off‐axis softening was introduced starting at 5 cm off‐axis (1–2.5 cm less water equivalent thickness in flattening filter). For the 6 MV FFF model, the fluence at 0.5 and 1.0 MeV was increased by 2% and 13%, respectively, and the beam profile corrections were decreased by 2%–6% starting at 13 cm off‐axis to better match the shoulders in the beam profiles. The shift in energy spectrum to lower average energy is consistent with literature.^13^ Calibration was performed according to IAEA TRS‐398^25^ formalism, in order to get 1MU = 1 cGy at 10 cm depth, for source‐surface distance of 90 cm and a reference field of 10 × 10 cm^2^ defined at 100 cm source‐axis distance.

While tuning the fit of the beam models to the measurements, we also took into account output factors, PDDs, and profiles for very small fields down to 0.6 × 0.6 cm^2^. In order to accurately simulate such small fields, a small voxel size of 0.1 × 0.1 × 0.1 cm^3^ was used for the dose calculation grid, unless indicated otherwise in Table 1.

**TABLE 1:** Complete overview of treatment fields and comparison metrics in study.

Phantom	Collection	Treatment fields	Comparison metric
Water tank	Rectangular treatment fields including small fields	0.6 × 0.6, 1 × 1, 2 × 2, 3 × 3, 4 × 4, 5 × 5, 8 × 8, 10 × 10, 15 × 15, 20 × 20, 30 × 30, 40 × 40, 5 × 20, 20 × 5 cm^2^ and diagonal 40 × 40 cm^2^	Output factors^a^, PDDs, dose profiles^e^
	Collimator rotations small fields^b^	0.6 × 0.6, 1 × 1, 0.6 × 5 cm^2^, at collimator angles 0°, 30°, 45°, 60°, 90°	Output factors, dose profiles^e^
	Off‐axis treatment fields^c^	10 × 10 cm^2^, offset by 5 cm in several directions in the X,Y plane	Output factors
	Small off‐axis treatment fields^a^	0.6 × 0.6 cm^2^, offset by 0, 3.75, and 11.25 cm in the X and/or Y directions	Dose profiles^e^
	Geometrical test fields^b^	H‐field, triangle, dumbbell, asymmetrical dumbbell, C‐field	Output factors, dose profiles^e^
ArcCHECK	Rectangular fields	0.6 × 0.6, 1 × 1, 2 × 2, 3 × 3, 5 × 5, 10 × 10, 20 × 20, 1 × 3, 2 × 5, 4 × 10, 12 × 30 cm^2^	Gamma pass rate, mean gamma index
	Clinical VMAT plans^d^	6 MV: 3 x head‐and‐neck, glioma, brain; 6 MV FFF: 2 x vertebra, 3 x stereotactic lung, 3 x multiple brain metastases	Gamma pass rate, Mean gamma index
Film dosimetry	Collimator rotations small fields^b^	0.6 × 0.6, 1 × 1, 0.6 × 5 cm^2^, at collimator angles 0°, 30°, 45°, 60°, 90°	Dose profiles^e^

^a^ MC calculated at 0.1% uncertainty.

^b^ MC calculated at 0.2% uncertainty.

^c^ Calculated with a 0.2 × 0.2 × 0.2 cm^3^ dose grid voxel size and 0.1% uncertainty for MC calculations.

^d^ Optimized with CC. For the purpose of this study, they have been additionally recalculated with MC.

^e^ Dose profiles in X and Y directions, coordinate system according to IEC 61217.

MC dose calculations were performed using an uncertainty setting of 0.3%, unless indicated otherwise in Table 1, in which case a lower value is used. This value refers to the standard deviation of voxel dose as a percentage of the maximum treatment setup dose *D*_max_, averaged over all voxels having a dose above 50% of *D*_max_. The relatively low value of 0.3% was chosen so that the statistical uncertainty causes minimal degradation of the fit to measurements while still obtaining reasonable calculation times. In clinical practice, a higher value might be acceptable. All final dose calculations were performed on a NVIDIA Quadro RTX 6000 GPU with the driver version 563.25 (NVIDIA Corp, Santa Clara, California, USA).

### 2.2 Validation tests

The tests can be divided into three categories corresponding to three phantoms: test fields irradiated in a water tank phantom (Blue phantom water tank; Wellhöfer Dosimetrie, Swarzenbruck, Germany), test fields and clinical treatment plans irradiated on an ArcCHECK phantom (Sun Nuclear Corporation, Melbourne, Florida, USA) and test fields irradiated on a film dosimetry phantom (PTW IMRT verification phantom, Freiburg, Germany). Table 1 provides a complete overview of all the treatment fields that were measured and calculated, as well as which comparison metrics were used.

#### 2.2.1 Water tank

Rectangular fields, collimator rotations, off‐axis treatment fields, and geometrical test fields were measured and calculated using the water tank phantom.

A stereotactic field diode (SFD) (Scanditronix Medical AB, Uppsala, Sweden) and CC13 ionization chamber (Wellhöfer Dosimetrie, Swarzenbruck, Germany) were used for measuring PDDs of smaller fields (≤ 4 × 4 cm^2^) and larger fields (> 3 × 3 cm^2^), respectively. For profiles and output factors, a linear diode array (LDA‐99SC, IBA, Louvain‐la‐Neuve, Belgium) or the SFD in water was used. According to IAEA TRS 483 recommendations,^8^ measurements of output factors for larger fields (> 3 × 3 cm^2^) were performed using the CC13 ionization chamber, and for smaller fields (2 × 2, 1 × 1 and 0.6 × 0.6 cm^2^), the SFD was used. Gul et al. have experimentally confirmed that the SFD can be used to measure output factors of small fields with the correction factors provided by IAEA TRS 483 recommendations.^26^

Output factors for these small fields were measured and calculated for a range of collimator angles to study how the output changes when rotating the collimator (Table 1). All output factors were measured at a depth of 10 cm in the center of each field and calculated in relation to the reference 10 × 10 cm^2^ field. The deviation between the measured and calculated output factors are reported as a percentage, which allows for a quantitative comparison of MC and CC. Dose profiles are measured for a selection of test fields—measurement and calculation results are plotted together in one figure for qualitative comparison and both normalized at the isocenter at 10 cm depth.

#### 2.2.2 ArcCHECK validation

The ArcCHECK device is a cylindrical acrylic phantom with a three‐dimensional array of 1386 diodes. The array has a helical configuration with 1 cm inter‐diode spacing, 1 cm pitch and 21 cm diameter, and an active detector size of 0.8 × 0.8 mm^2^ [ArcCHECK User's Guide (Sun Nuclear Corporation 2020)]. The diode array is located at a physical depth of 2.9 cm. The device can be used with or without a cavity insert that fills the cylindrical air cavity in the device, turning it into a mostly solid phantom. During measurements, the ArcCHECK reads out the accumulated diode charge every 50 ms. To perform ArcCHECK analysis, the software package *SNC Patient* (Sun Nuclear Corporation, version 8.4.1.2) was used to import a DICOM RT Dose file and an ArcCHECK measured file for comparison. The import filter extracts a cylindrical dose plane from the imported 3D volume of the RT Dose file for 2D dose comparison with the ArcCHECK diodes.

**FIGURE 1:**
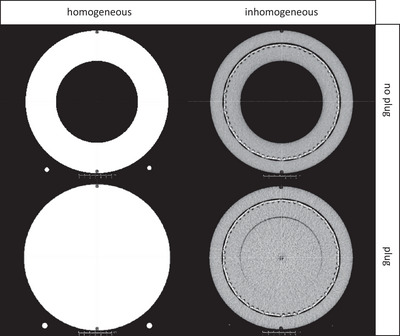
Four images of the ArcCHECK phantom. Left column: “homogeneous” artificial images without (top) and with (bottom) cavity plug. Right column: “inhomogeneous” MVCT images without (top) and with (bottom) cavity plug.

In order to perform TPS calculations for dose verification, megavoltage computed tomography (MVCT) images (Figure 1, right column) and a CT‐density (electron density) conversion file from MVCT were provided by Sun Nuclear, as well as artificial homogeneous images that feature only two distinct densities for the two materials: air and PMMA (1.18 g/cm^2^) (Figure 1, left column). According to Sun Nuclear recommendations, in our clinical practice, the ArcCHECK is used without cavity plug and the corresponding inhomogeneous MVCT image is used for TPS calculations.^18^ In this study, on the other hand, all four configurations (with/without plug, homogeneous/inhomogeneous) were studied. For analysis on the homogeneous phantom, measurement data were converted by applying heterogeneity correction factors provided by Sun Nuclear. For 6 MV FFF, no heterogeneity correction factors for VersaHD were made available by Sun Nuclear for the ArcCHECK without plug. Therefore, no measurements were done in this setting. For clinical VMAT plans, a gantry spacing of 2° was used, and couch angles were collapsed to 0°.

Notably, the RayStation MC dose engine reports results in dose‐to‐medium (*D*_m_),^21^ whereas the absolute dose calibration for ArcCHECK is done in a water tank, signifying a dose‐to‐water (*D*_w_) reference condition. A scaling factor *D*_m_/*D*_w_ was applied to convert the calculated MC dose to *D*_w_ to allow for comparison with the measurements.^27^ The scaling factors (for 6 and 6 MV FFF) for PMMA was obtained empirically by varying the scaling factor to optimize the fit between ArcCHECK measurements and calculations for three medium‐sized fields (5 × 5, 4 × 10, 10 × 10 cm^2^). CC reports in *D*_w_^21^ and therefore did not require a scaling factor.

Nowadays, strict gamma analysis criteria are generally recommended by the scientific community for patient specific QA: 3%/2 mm.^28^ Gamma pass rates and mean gamma values are reported for ArcCHECK results using two sets of criteria for global dose difference and distance to agreement (DTA): 3%/2 mm and 2%/1 mm.^29^, ^30^ In both cases, a 10% threshold proposed by the AAPM Task Group 119^31^ was used for the minimum dose percent value for the point to be included in the analysis. The smallest static fields that irradiate less than five diodes above the 10% threshold (the 0.6 × 0.6 cm^2^ and 1 × 1 cm^2^ fields) will not be used for comparing dose engines, because the small number of diodes will not produce reliable mean gamma values or pass rates. The results will be tabulated in the supplementary materials.

To calculate gamma indices, SNC Patient software interpolates the TPS data. Initially, the interpolation is made to a 0.1 × 0.1 cm^2^ grid, but further interpolation may be performed when searching for the DTA (*SNC Patient* Software Help, Sun Nuclear Corporation).

For clinical plans, a gamma pass rate above 95% at 3%/2 mm is considered clinically acceptable. A pass rate of 90%−95% is also acceptable if the gamma failure distribution indicates that the deviations are clinically irrelevant in location and magnitude.^28^ For the simple rectangular fields, a gamma pass rate above 95% for most fields is expected, as these fields should be easy to model accurately for any dose engine. Mean gamma values will also be reported, since they are a more sensitive measure for comparing dose engines than pass rates.

The *SNC patient* software was also used to perform a gamma analysis, comparing two sets of calculations (MC vs. CC), in order to visualize and quantify dose differences. In this case, a threshold of 0% is used to study all dose differences, including in low‐dose regions.

As detailed in Table 1, ArcCHECK analysis was performed for static rectangular fields as well as 13 anonymized clinical VMAT plans. These 13 plans were optimized with the clinical CC model. For the MC calculation result, only the final dose was recalculated with MC (no optimization step was performed).

#### 2.2.3 Film dosimetry

To measure the effect of collimator rotations on the dose output of very small fields, film dosimetry was used as an additional method, complementary to the measurements with the SFD.

Absolute film dosimetry was carried out with Gafchromic EBT3 films (International Specialty Products, USA). For calibration and measurements, EBT3 films were placed in a PTW acrylic phantom (30 × 30 × 10 cm^3^). After irradiation, 24‐h waiting time was used before films were scanned in the same direction with EPSON V750 PRO color scanner and analyzed using DoseLab 4.11.^32^, ^33^

## 3 RESULTS

### 3.1 Water tank

#### 3.1.1 Rectangular fields, geometrical test fields

**FIGURE 2:**
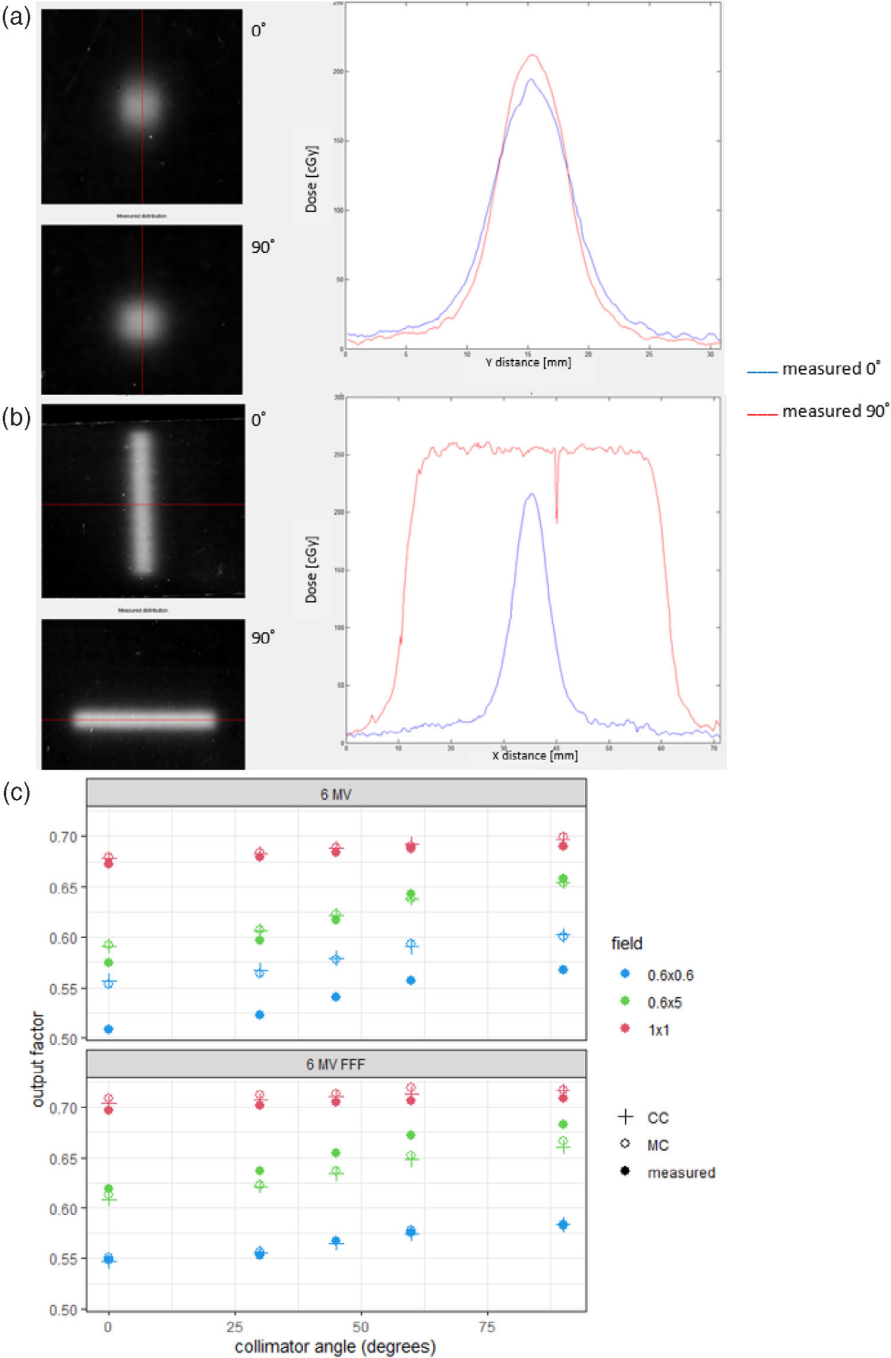
Output factors as a function of collimator angles. (a) 6 MV EBT3 film measurements of 0.6 x 0.6 cm^2^ for 0˚ (blue curve) and 90˚(red curve) collimator angle; (b) EBT3 film measurements of 0.6 x 5.0 cm^2^ for 0˚ (blue curve) and 90˚ (red curve); (c) comparison of measured output factors to TPS calculations (CC and MC) as a function of collimator angle for 6 MV and 6 MV FFF.

**FIGURE 3:**
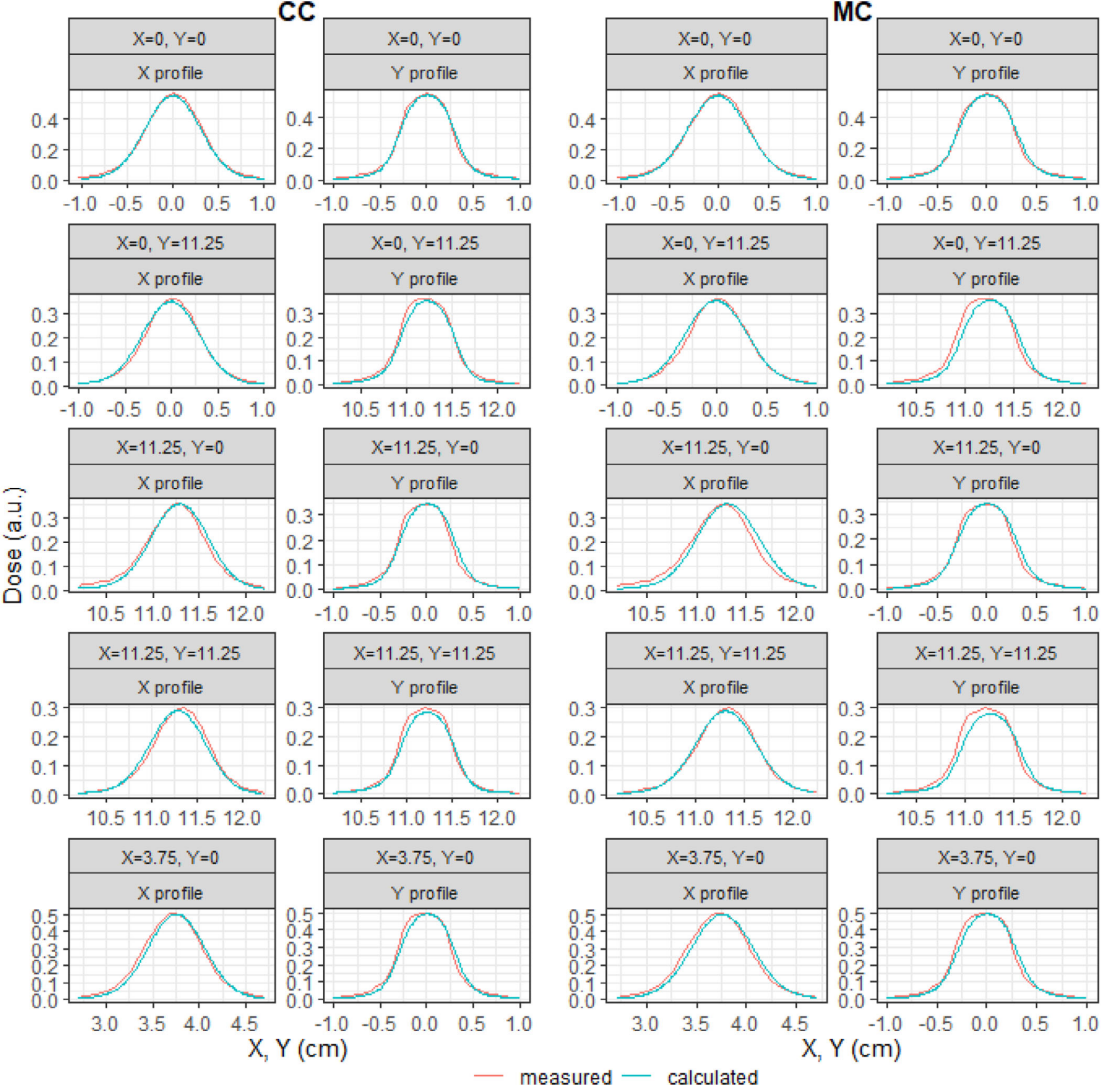
Comparison of measured and calculated dose profiles for off‐axis 0.6 × 0.6 cm^2^ fields. The left two columns show X and Y profiles calculated with CC. The right two columns show X and Y profiles calculated with MC. Each row of plots represents a field; the label above each plot indicates the center of the field in cm. Red lines are measured data, blue lines are calculated.

Water tank verification results are presented in the Supplementary materials, in Figures 1A–3A, and Tables 1A–6A.

The measured and calculated dose profiles and PDDs of rectangular fields (Figure 1A, 2A) show that the 6MV models (both CC and MC) overall fit better to measurements than the 6MV FFF models, in particular the PDDs for the two smallest fields.

A comparison between measured and calculated output factors, averaged over all rectangular static fields, showed a (root mean squared) deviation of 0.4%–0.6% (CC) and 0.6% (MC) (Table 1A, 2A). The difference in this deviation between CC and MC is clinically negligible (< 0.2%) and also includes statistical noise in the MC calculations.

Similarly, output factors for off‐axis medium‐sized fields show that the average MC‐computed deviation between measurements and calculations is 0.3% (6 MV) and 0.2% (6MV FFF) higher than for CC. For most fields, the difference between the measured and calculated output factors is within 1% for both dose algorithms.

Tables 5A and 6A present measured and calculated output factors for five geometrical test fields. For 6 MV, all differences between measurement and calculation are within 3%. For 6 MV FFF, all differences are well within 2%. Averaged (root mean square) over all fields, the two dose engines produce comparable results—the averaged MC‐computed difference is 0.2% lower compared to CC.

To summarize, for water tank measurements of these rectangular and geometric test fields, the goodness of fit to the measurements of the CC and MC models is considered to be equivalent.

#### 3.1.2 Collimator angle dependence for small fields

For 6 MV and 6 MV FFF, the collimator angle dependence was measured with an SFD at our VersaHD accelerator, as well as film dosimetry to verify our findings. For 6 MV, we measured a dose increase of 12% for the 0.6 × 0.6 cm^2^ field and of 15% for 0.6 × 5 cm^2^ field with the collimator rotation from 0° to 90°. Similar results were found with film measurements (Figure 2). This effect of collimator angle dependence was a reason for us to use an output factor of 45° instead of 0° for the 0.6 × 0.6 cm^2^ field in RayStation for 6 MV for both CC and MC. Additionally, a reason to choose for the output factor of 45° was that this collimator angle is used in our clinical VMAT plans, except for multiple metastases plans. Both CC and MC models in RayStation can reproduce this behavior: a dose increase of 8% (CC) and 9% (MC) for the 0.6 × 0.6 cm^2^ field and of 11% (CC) and 10% (MC) for 0.6 × 5 cm^2^ field with the collimator rotation from 0° to 90°. For the 1 × 1 cm^2^ field, a dose increase is only 1% with the collimator rotation from 0° to 90° in measurements and TPS calculations. For square fields larger than the 1 × 1 cm^2^, the effect is negligible. We measured a larger dose dependence of the collimator angle for 6 MV than for 6MV FFF. For 6MV FFF, a dose increase of 6% for the 0.6 × 0.6 cm^2^ field and of 10% for 0.6 × 5 cm^2^ field with the collimator rotation from 0° to 90° was measured. The CC and MC models in RayStation can reproduce this dependence on collimator angle very well: a dose increase of 7% (CC) and 6% (MC) for the 0.6 × 0.6 cm^2^ field and of 9% (CC and MC) for 0.6 × 5 cm^2^ field with the collimator rotation from 0° to 90°.

#### 3.1.3 Small off‐axis fields

Dose profiles of small (0.6 × 0.6 cm^2^) off‐axis fields show good agreement between measurements and calculations for both CC and MC (Figure 3). A close inspection shows that with MC, the off‐axis dose profiles are shifted away from the isocenter, relative to CC. These shifts are smaller than 1 mm.

### 3.2 ArcCHECK

#### 3.2.1 Rectangular fields

**FIGURE 4:**
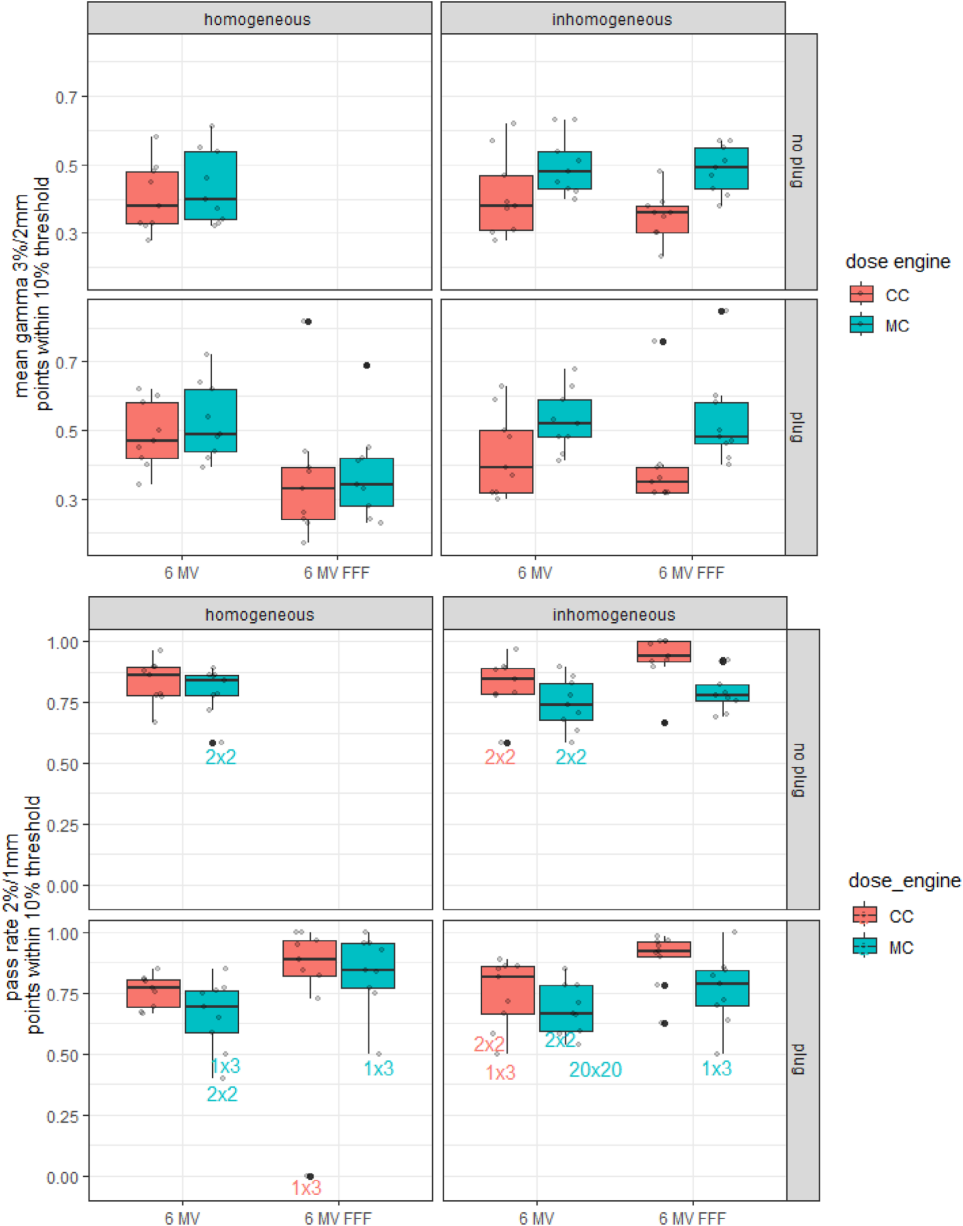
ArcCHECK results for a collection of nine rectangular fields, presented as box‐and‐whiskers plots grouped by dose engine, beam energy, presence of cavity plug and image homogeneity. Top: Mean gamma values at 3%/2 mm dose criteria; bottom: gamma pass rates at 2%/1 mm dose criteria.

Mean gamma results at the 3%/2 mm dose criteria, and at the 2%/1 mm dose criteria for the inhomogeneous virtual phantoms are generally higher for MC compared to CC, as well as compared to MC calculations on the homogeneous phantom (Figure 4, top panel of four plots). Within the homogeneous phantom, the mean gamma results are more equal between CC and MC (Figure 4 top panel, left column).

**FIGURE 5:**
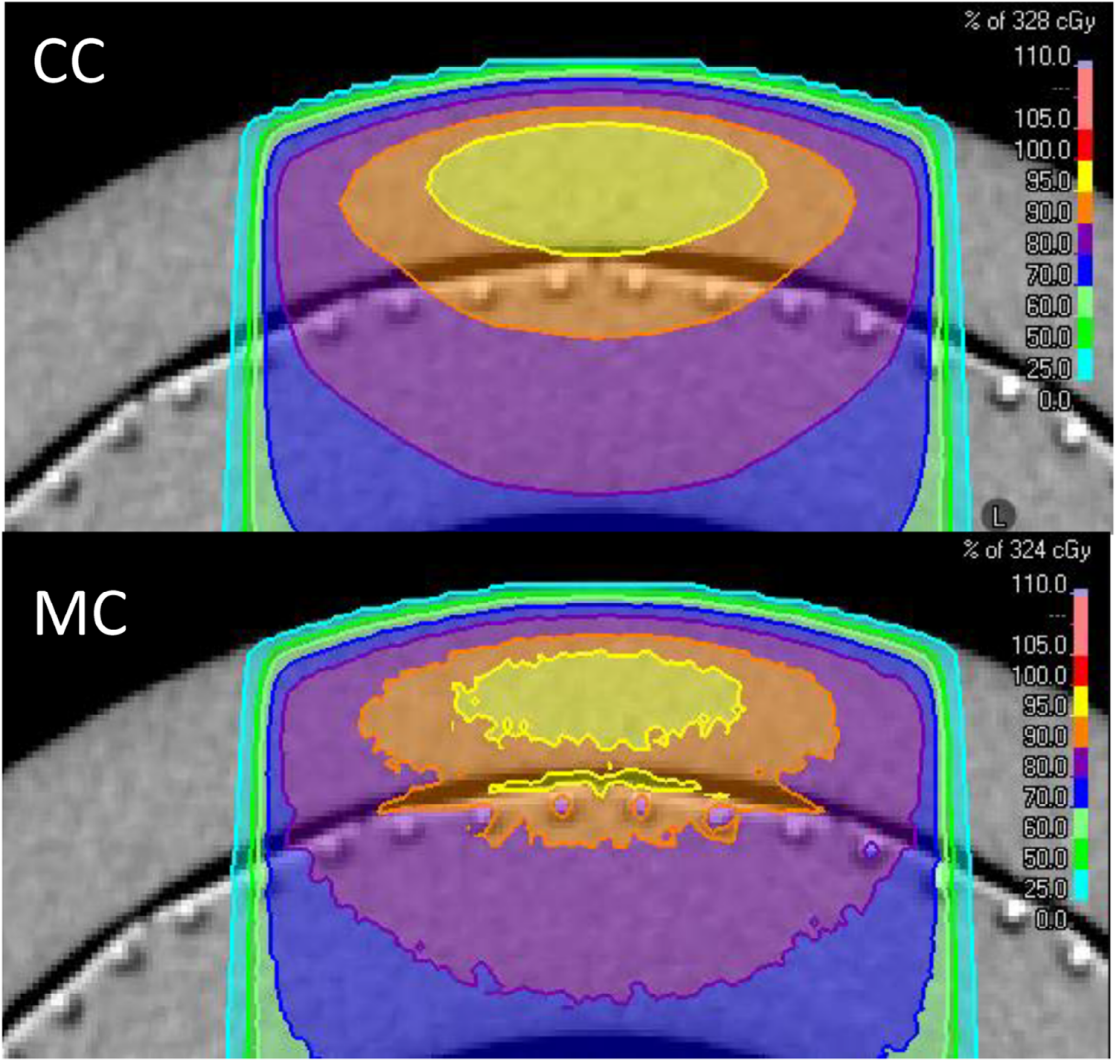
Cross sections of calculated dose distributions of a 10 × 10 cm^2^ treatment field on the inhomogeneous ArcCHECK image with plug. Top: CC algorithm; bottom: MC algorithm.

Cross‐sections of the dose distribution calculated by CC and MC for the inhomogeneous ArcCHECK phantom configuration show that the MC dose distribution is highly sensitive to local density variations around the diodes (Figure 5), resulting in local dose differences > 5%. This introduces additional uncertainties to the fit between measurement and simulation. In contrast, the CC dose distribution shows practically no response to these local heterogeneities.

In agreement with the relatively poor gamma results of the inhomogeneous phantoms in Figure 4, the homogeneous configuration with cavity plug is considered the most suited for Monte Carlo calculations in our situation. For this preferred configuration, gamma pass rates for all rectangular fields are presented in Table 2. The pass rates at 3%/2 mm are well above 90% and mostly above 95% for all fields except the smallest (0.6 × 0.6, 1 × 1, 2 × 2, and 1 × 3 cm^2^), which irradiate a very small number of diodes, as well as the largest field (20 × 20 cm^2^).

**TABLE 2:** ArcCHECK results for rectangular fields in the homogeneous configuration with cavity plug. Presented are the gamma pass rate at 3%/2 mm (γ_3,2_); gamma pass rate at 2%/1 mm (γ_2,1_); and the number of diodes above the 10% threshold for gamma analysis (*n*).

	6 MV						6 MV FFF					
	CC			MC			CC			MC		
Field (cm^2^)	Pass rate γ_3,2_ (%)	Pass rate γ_2,1_ (%)	*n*	Pass rate γ_3,2_ (%)	Pass rate γ_2,1_ (%)	*n*	Pass rate γ_3,2_ (%)	Pass rate γ_2,1_ (%)	*n*	Pass rate γ_3,2_ (%)	Pass rate γ_2,1_ (%)	*n*
0.6 × 0.6	100	100	2	100	100	2	0	0	1	0	0	1
1 × 1	33.3	0	3	33.3	0	3	66.7	33.3	3	67	0	3
2 × 2	80	40	15	84.6	69.2	13	100	84.6	13	100	84.6	13
3 × 3	100	76	25	100	80	25	100	92.9	28	100	88.9	27
5 × 5	100	77.3	66	100	80.3	66	100	95.5	66	100	100	66
10 × 10	94.5	69.4	235	96.2	75.7	235	100	83.9	224	100	95	222
20 × 20	86.4	58.6	1330	82.7	67.3	1329	92.4	74.8	1236	95.1	72.7	1225
1 × 3	66.7	50	6	83.3	66.7	6	66.7	50	6	77.8	0	9
2 × 5	100	85.2	27	100	85.2	27	100	100	27	100	100	27
4 × 10	99	74.7	99	98	80.8	99	100	95.6	91	100	96.7	91
12 × 30	92.6	64.8	861	92.1	77.4	861	96.8	77.1	821	97.7	82.4	819

#### 3.2.2 Clinical treatment plans

**FIGURE 6:**
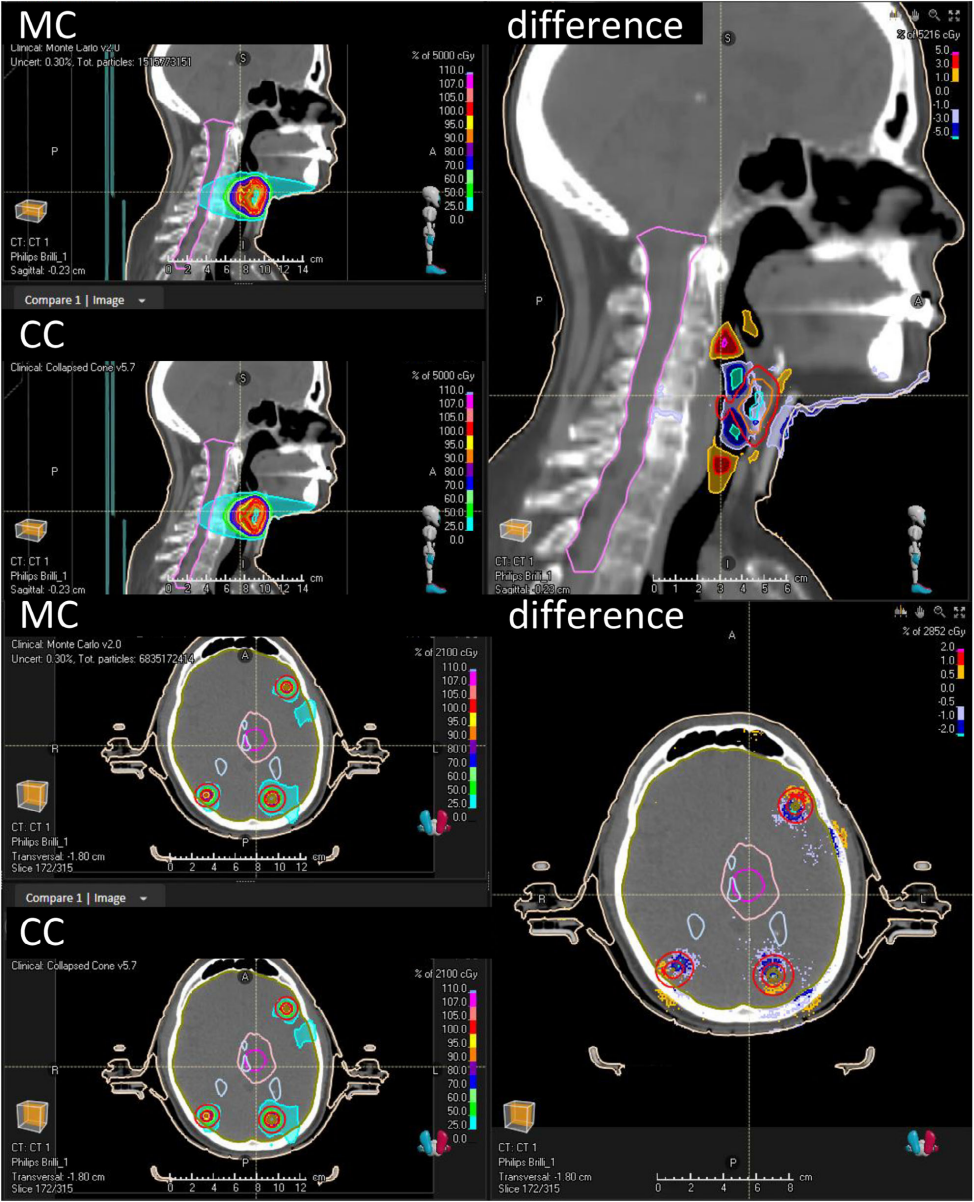
Comparisons between Monte Carlo‐calculated dose and Collapsed Cone‐calculated dose for two clinical VMAT plans. Top panel: head‐and‐neck plan. Bottom panel: multiple brain metastases plan (eight metastases). Both panels contain three windows: top left is the MC dose, bottom left is CC dose, the bigger panel on the right is the dose difference in % of the maximum dose. A positive difference means the MC dose is higher than the CC dose.

To illustrate the dose differences that can be expected in clinical treatment plans, two comparisons between a MC‐calculated dose and a CC‐calculated dose are shown (Figure 6). In the head‐and‐neck plan, differences in dose exceeding 5% of the maximum dose are observed in and around air‐filled structures. Such differences due to heterogeneities in the patient model will not be reproduced in ArcCHECK analysis because the patient model is not included in the analysis. In the multiple brain metastases plan, dose differences of 1%−2% of the max dose, both positive and negative, are observed in the high‐dose regions. These differences are not caused by heterogeneous structures, but rather by differences in dose algorithms. Such effects will be reproduced in ArcCHECK analysis. Note that these figures show a comparison between *D*_m_ and *D*_w_—in soft tissue, *D*_m_ is about 1% lower than *D*_w_.^34^

**FIGURE 7:**
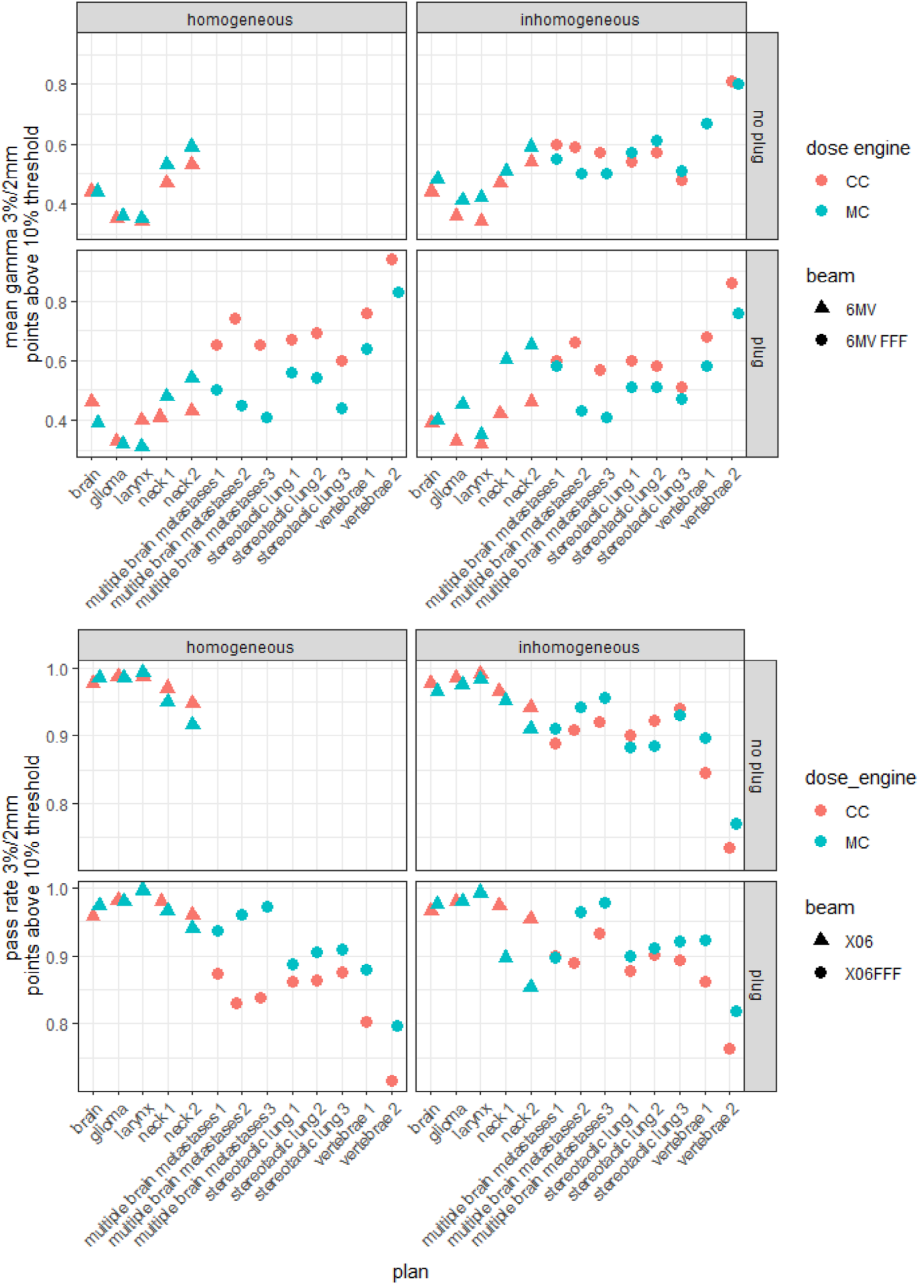
ArcCHECK results for 13 clinical treatment plans, grouped by dose engine, beam energy, presence of cavity plug and image homogeneity. Top: mean gamma value at 3%/2 mm dose criteria; bottom: pass rates at 3%/2 mm dose criteria.

For the 13 clinical treatment plans in the preferred homogeneous configuration of ArcCHECK with cavity plug, MC resulted in a lower mean gamma as compared to CC for all 6MV FFF plans (Figure 7, bottom left plot, blue versus red, −64% to −13% difference) and a majority of the 6MV plans (−29% to +20% difference). The calculations on an inhomogeneous phantom (Figure 7, right column) resulted in more equal results between MC and CC. Interestingly, this is in contrast to the results for the rectangular fields, where the inhomogeneous phantom resulted in consistently poorer gamma values for MC relative to CC.

**FIGURE 8:**
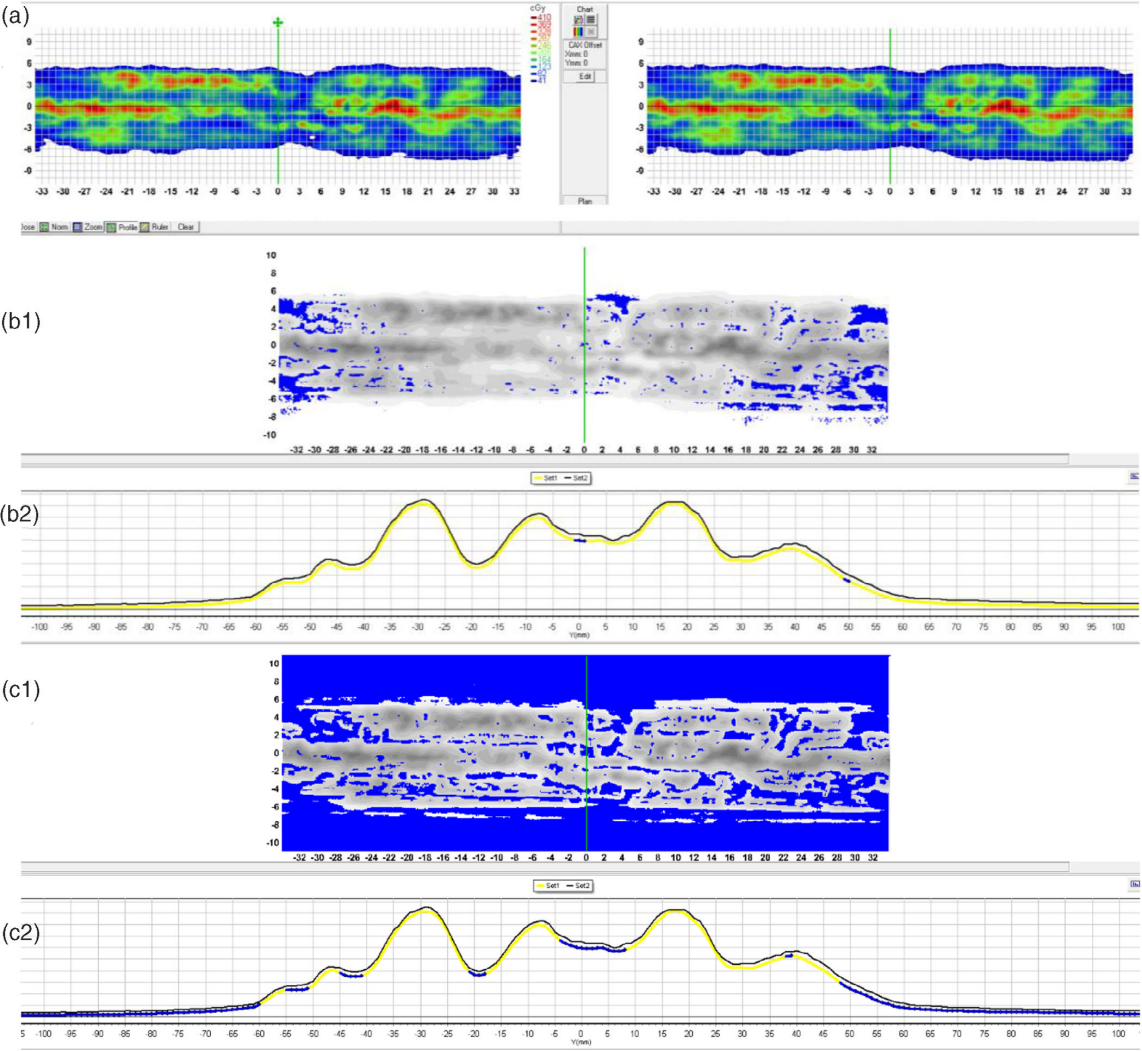
ArcCHECK analysis (homogeneous phantom with cavity plug) for the “multiple brain metastases 2″ treatment plan, comparing a CC and a MC calculation (no measurements). The gamma threshold is set to 0%. (a) Calculated dose distributions at the ArcCHECK diode interface, left is CC and right is MC. The grid represents the positions of the diodes, transformed from the helical configuration to a 2D rectangular grid. (b1) Global gamma analysis at 2%/2 mm. Blue dots indicate gamma‐failed points where the CC calculated dose is lower than MC. (b2) Y‐profile corresponding to the green line in B1. (c1) Local gamma analysis at 2%/2 mm. Blue dots indicate gamma‐failed points where the CC calculated dose is lower than MC. (c2) Y‐profile corresponding to the green line in C1.

A gamma analysis comparing two TPS simulations in *SNC Patient* for the *multiple brain metastases 2* plan is shown in Figure 8.

The MC‐calculated dose is higher than the CC‐calculated dose at every position, but mostly (> 90%) within the global 2%/2 mm criteria. The 2%/2 mm gamma criteria were chosen to clearly visualize the distribution of gamma fails, aiming for a global pass rate of 90%−95% and thereby highlighting the first 5%−10% of failing points. Looking at the regions where the local and global gamma criteria are not passed (Figure 8, panels b1 and c1), it is apparent that the biggest differences between MC and CC occur in the low‐dose regions. In these out‐of‐field regions, the MC‐calculated dose is higher than the CC‐calculated dose. The same pattern appears for all 13 clinical treatment plans.

## 4 DISCUSSION

This study includes a comparative analysis of two most common dose engines implementations in RayStation, providing new insights into the strengths and limitations of CC and MC algorithms. Two MC models were commissioned in‐house for 6 MV and 6 MV FFF photon beams. A number of verification tests on a water tank phantom, an ArcCHECK phantom and a film dosimetry phantom were performed in order to validate the performance of the MC model and compare it to the clinical CC model. For each verification test, TPS results using both CC and MC are obtained as well as a physical measurement on a LINAC. The agreement between measurement and calculation is compared between the two dose models using a water tank phantom as well as ArcCHECK analysis.

As expected, the water tank results with simple rectangular fields and off‐axis rectangular fields show comparable results between the CC and MC models in terms of PDDs and dose profiles for the symmetric rectangular fields and output factors for the off‐axis fields. These results simply indicate that the CC and MC models, both commissioned in‐house, are overall of comparable quality. A more detailed look into the PDDs and profiles showed that for 6 MV FFF, MC performs slightly worse than CC in PDDs and in‐field profiles, but better in Y‐jaw penumbras. These results are in good agreement with previously published.^13^

Measured output factors for the small fields with collimator rotations demonstrated a strong dependency of dose output with respect to collimator angle in small fields (0.6 × 0.6, 1.0 × 1.0, and 0.6 × 5 cm^2^) in the VersaHD treatment head with Agility MLC. To our knowledge, this has not been reported in literature yet. Interestingly, this effect is modelled well by both the CC and MC models. Our explanations for this dependence on collimator rotation for small fields are the oval shape of the primary source (and the secondary source for 6 MV), which results in the different penumbra for the collimator angles of 0° and 90°. Additionally, there is a difference between the MLC and block penumbra due to the different distance to the source.

From our measurements, we suggest that the combination of the MC dose algorithm and an inhomogeneous virtual ArcCHECK image might not be suitable. We recommend a homogeneous phantom image with cavity plug as the best configuration for performing ArcCHECK verification with MC for the 6 MV and 6 MV FFF VersaHD beams. A limitation of using MC is that when using a CT density table for dose calculations, a conversion between *D*_m_ and *D*_w_ is required, adding additional uncertainty to the analysis. This conversion can be avoided by applying a material override for PMMA based on water, but with a higher density to match the attenuation of PMMA.^35^ The correct density depends on the TPS and calculation algorithm [*SNC Patient* Software Help (Sun Nuclear Corporation)]. In our case, this resulted in comparable gamma index results to the homogeneous image case presented here, indicating that our empirical conversion factor was chosen well.

The poor ArcCHECK performance of MC on the inhomogeneous virtual phantoms that was observed for static rectangular fields was not as clear for the clinical treatment plans. In the clinical scenarios, there was no obvious difference between the homogeneous and inhomogeneous phantom, and the gamma results for MC were consistently better (lower mean gamma, higher passing rate) than the CC results. A potential explanation for this could be, firstly, that the issues regarding the heterogeneities around the diodes get averaged out in a VMAT plan, compared to a static field. Or alternatively, the effect is still present but is compensated by the advantage of MC being better able to model the highly modulated treatment fields in clinical plans. For the 13 clinical treatment plans in the preferred homogeneous configuration, MC resulted in a lower mean gamma as compared to CC for all 6 MV FFF plans (Figure 7, left column blue vs. red, −64% to −13% difference) and a majority of the 6 MV plans (−29% to +20% difference). Given that this overall advantage of MC was absent for simple rectangular fields but evident in the clinical treatment plans, it can be drawn that MC is likely to deliver superior performance over CC, particularly in the context of highly modulated treatment plans.

**FIGURE 9:**
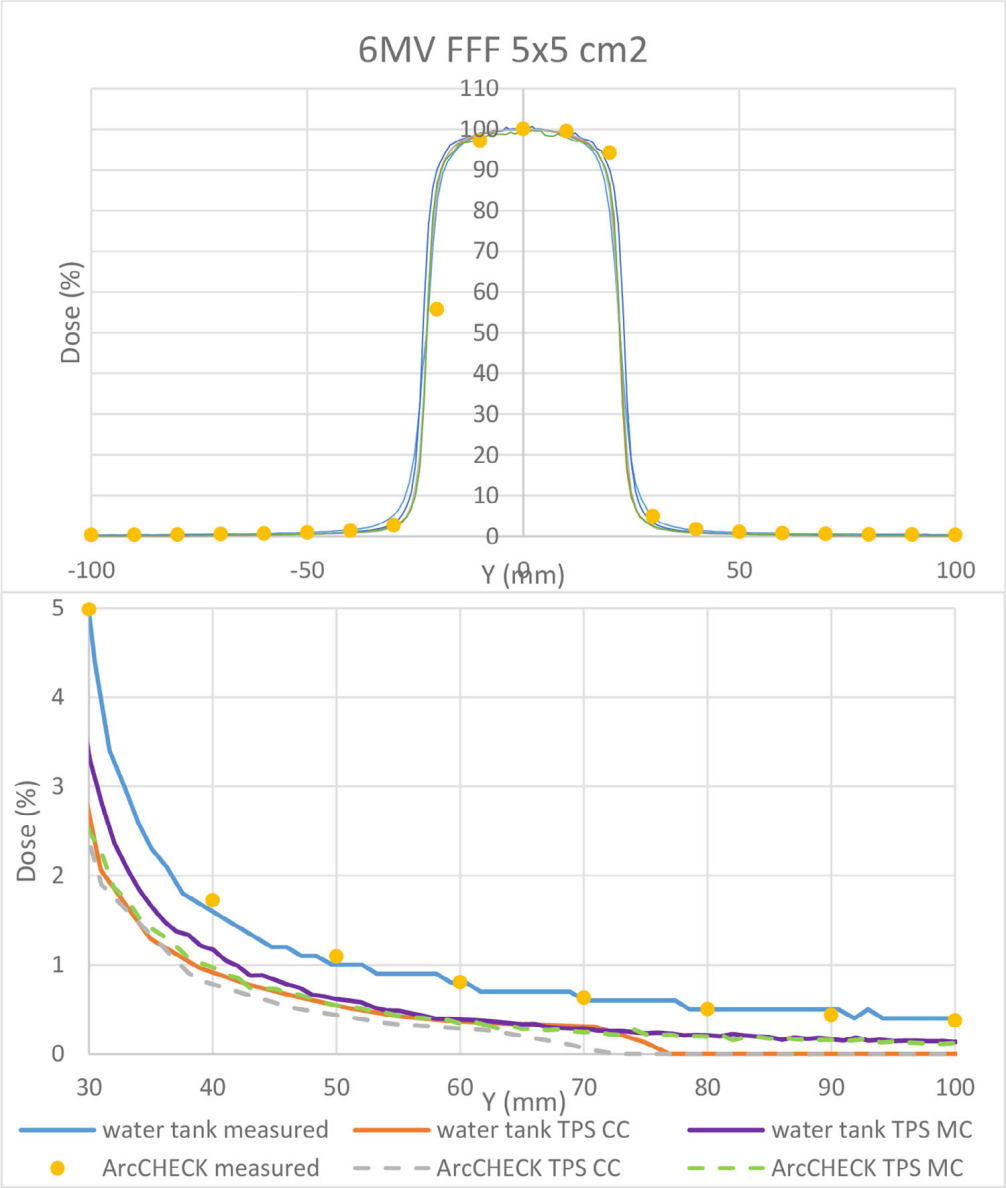
Measured and calculated (Collapsed Cone) results showing the effect of the calculation mask in the Collapsed Cone algorithm for 6 MV FFF: calculated dose profiles are cut off around 7 cm off‐axis. The bottom plot shows a zoomed in region of the top plot.

To better understand the differences found between MC and CC dose distributions, an additional gamma analysis was performed comparing the two ArcCHECK calculation results. These showed that for all clinical plans, the MC‐calculated dose was higher than the CC‐calculated dose for all plans, especially in the out‐of‐field regions. This can be explained by the following limitation of the CC algorithm. In the CC algorithm, a calculation mask is applied around the TERMA region when there is no TERMA above 0.5% of the maximum TERMA within 5 cm radiological distance along the ray.^21^ This results in better computation times, at the cost of the dose calculation being cut off out‐of‐field. In order to illustrate this effect, Figure 9 shows that CC calculated dose profiles are cut off around 7 cm off‐axis. This effect has been examined in our clinic before (Figure 9). The MC algorithm does not have this limitation, leading to a higher (and more accurate) dose calculation out‐of‐field. This small effect can add up for highly modulated treatment plans with small field sizes and a large number of beams, which could explain that the calculated MC dose is higher than the CC dose out‐of‐field across all treatment plans.

Another approximation in CC that affects off‐axis dose is the no‐kernel tilt approximations.^21^ This approximation pulls the dose more toward the isocenter at off‐axis positions, which can be seen in the dose profiles of small off‐axis fields. This effect can be compensated in RayStation by applying off‐axis parameters (*offset*, *gain*, and *curvature*) that correct the position of Y‐jaws or MLC leaves. Both our CC and MC models have the same off‐axis parameters, which were optimized for CC but not for MC. The MC model could potentially be tuned closer to the measured data by setting the MLC gain and offset to zero.

It is well known that the MC‐based dose algorithms are better suited to calculate dose distributions in heterogeneities as compared to CC algorithms. This has been verified many times in literature^13^, ^14^, ^16^ for RayStation and is not the focus of this study, except for the inhomogeneous ArcCHECK phantom. In this study, we have demonstrated that even in homogeneous phantoms (water tank, homogeneous ArcCHECK), our MC model can outperform CC to a clinically relevant extent, most notably seen in the ArcCHECK results for 13 clinical VMAT plans. For more simple treatment configurations such as static rectangular fields, no notable difference in performance was seen between the two dose algorithms.

## 5 CONCLUSION

Our study reveals a strong dependency of dose output to collimator angle for VersaHD with Agility MLC in very small or narrow fields, effectively reproduced by both CC and MC dose algorithms in RayStation.

Our findings highlight a suboptimal agreement between measurements and MC calculations for simple rectangular fields when utilizing inhomogeneous ArcCHECK images. Therefore, we advocate for the use of homogeneous phantom images, particularly for static fields, in ArcCHECK verification with MC.

For simple rectangular fields, neither MC nor CC demonstrated a performance advantage, a consensus supported by both water tank and ArcCHECK results. The analysis of ArcCHECK results from clinical treatment plans indicates comparable performance between our MC and CC models for 6 MV. However, for 6 MV FFF, MC consistently outperformed CC, with ArcCHECK results exhibiting a notable improvement (13%−64% lower mean gamma index). This suggests a potential superiority of MC over CC not explained by heterogeneous structures, but by small fields.

## AUTHOR CONTRIBUTIONS

Anna L. Petoukhova conceived the study and supervised the project. The manuscript was drafted primarily by Guus B. Spenkelink and Anna L. Petoukhova, with some sections co‐authored by Sophie C. Huijskens. Anna L. Petoukhova and Guus B. Spenkelink commissioned the Monte Carlo beam model for simulations. Guus B. Spenkelink, Sophie C. Huijskens, Marc de Goede, Wilhelmus J. van der Star, and Jaap van Egmond performed experiments and analyzed data. Guus B. Spenkelink and Sophie C. Huijskens performed simulations. Jaap D. Zindler contributed to the discussion, provided critical feedback from a medical point of view, and provided patients’ clinical treatment plans. All authors discussed the results and contributed to the final manuscript.

## CONFLICT OF INTEREST STATEMENT

The authors declare no conflicts of interest.

## Supporting information



Supporting information
