# Supplementary material for: Comparative assessment and QA measurement array validation of Monte Carlo and Collapsed Cone dose algorithms for small fields and clinical treatment plans
Source: J Appl Clin Med Phys. 2024 Sep 17;25(12):e14522. doi: 10.1002/acm2.14522 (PMC11633799; doi:10.1002/acm2.14522)
Supplement: Supplementary file 1 — Supporting information [file ACM2-25-e14522-s001.pdf]

SUPPLEMENTARY DATA

WATER TANK RESULTS

Rectangular fields

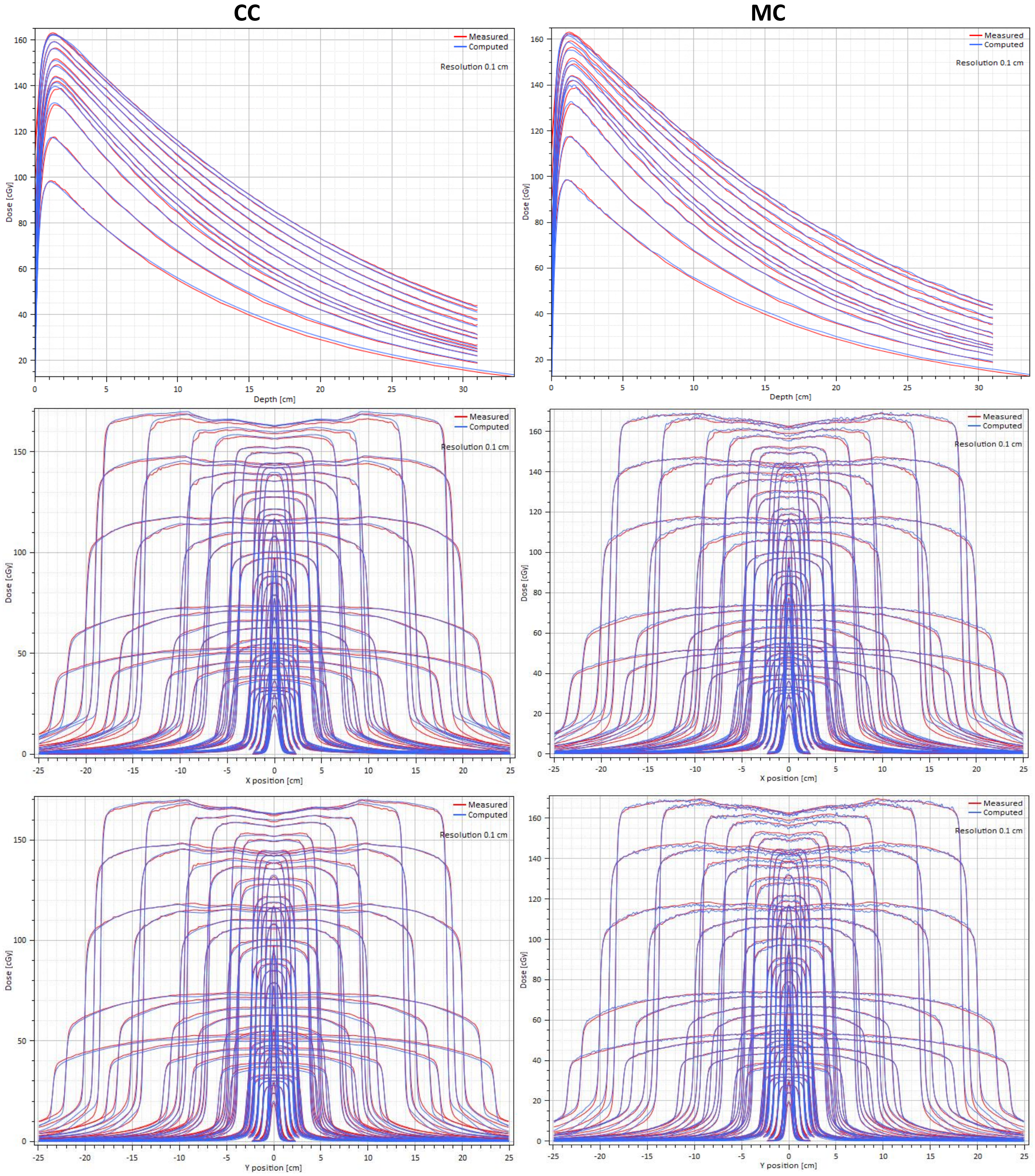

Figure 1A. Percentage depth dose curves (upper row), X-profiles (middle row) and Y-profiles (bottom row) for the 6 MV beam model. Measured data are shown in red, calculated data are shown in blue. Left column: Collapsed Cone; right column: Monte Carlo. The curves within each plot represent 12 different field sizes, from 0.6x0.6 cm^2 to 40x40 cm^2. Additionally, in the dose profiles all fields sizes are shown at 5 separate depths.

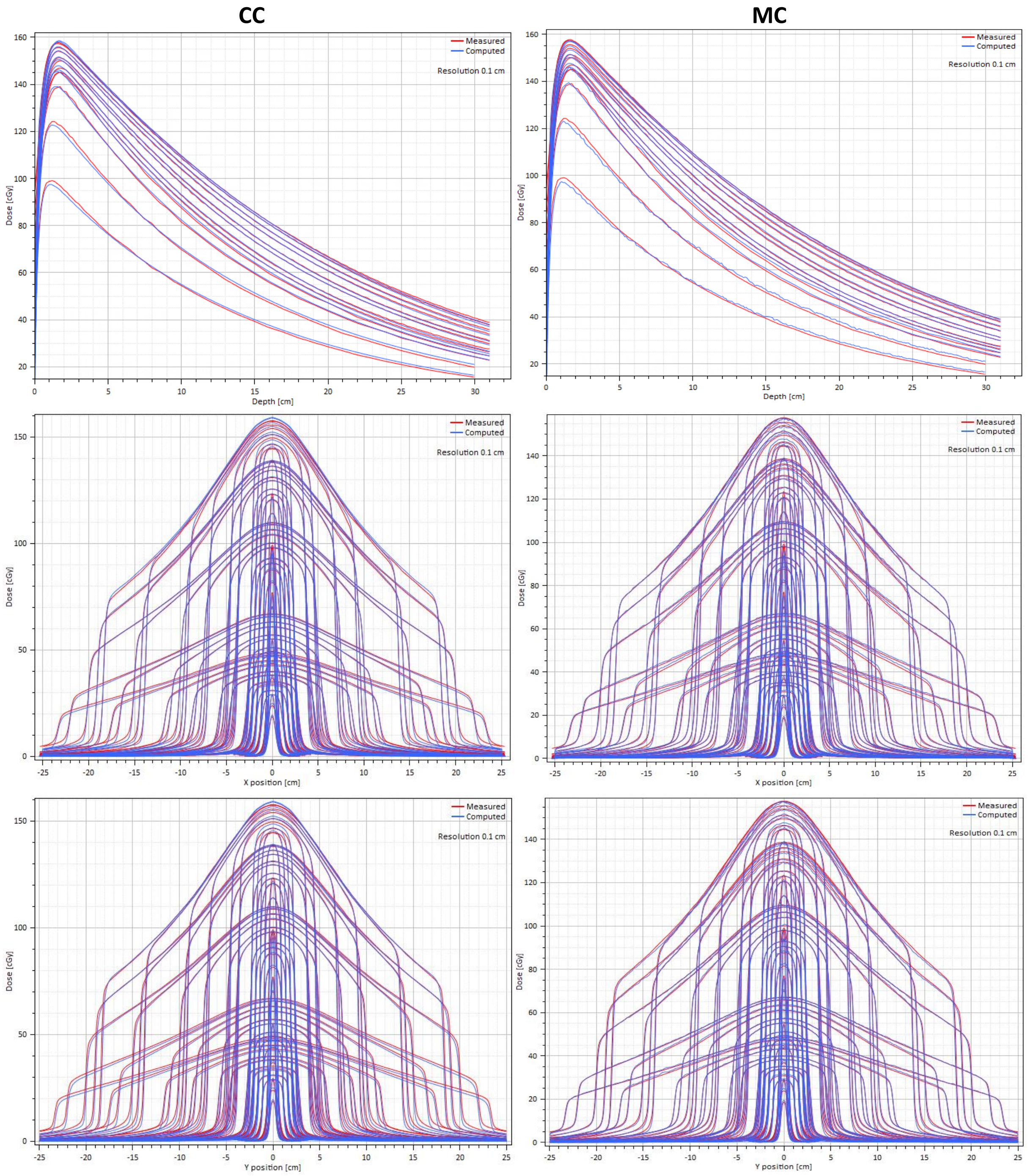

Figure 2A. Percentage depth dose curves (upper row), X-profiles (middle row) and Y-profiles (bottom row) for the 6 MV FFF beam model. Measured data are shown in red, calculated data are shown in blue. Left column: Collapsed Cone; right column: Monte Carlo. The curves within each plot represent 12 different field sizes, from 0.6x0.6 cm^2 to 40x40 cm^2. Additionally, in the dose profiles all fields sizes are shown at 5 separate depths.

Table 1A: Output factors for 6 MV rectangular fields in a water tank

field size (cm²)	output factor measured	output factor CC	output factor MC	difference CC	difference MC
1x1	0.674	0.678	0.676	0.5%	0.3%
2x2	0.789	0.787	0.787	-0.3%	-0.3%
3x3	0.847	0.851	0.845	0.4%	-0.2%
4x4	0.880	0.881	0.883	0.1%	0.3%
5x5	0.907	0.907	0.909	0.0%	0.1%
8x8	0.970	0.975	0.974	0.5%	0.4%
10x10	1.000	1.006	1.006	0.6%	0.7%
15x15	1.058	1.064	1.056	0.6%	-0.1%
20x20	1.095	1.100	1.089	0.5%	-0.6%
30x30	1.141	1.144	1.156	0.3%	1.4%
40x40	1.160	1.163	1.170	0.2%	0.9%
5x20	0.962	0.962	0.959	0.1%	-0.3%
20x5	0.969	0.971	0.963	0.2%	-0.6%
			root mean squared:	0.40%	0.58%

Table 2A. Output factors for 6 MV FFF rectangular fields in a water tank

field size (cm²)	output factor measured	output factor CC	output factor MC	difference CC	difference MC
1x1	0.711	0.704	0.706	-1.0%	-0.7%
2x2	0.815	0.824	0.825	1.1%	1.3%
3x3	0.875	0.882	0.880	0.8%	0.5%
4x4	0.905	0.908	0.907	0.3%	0.2%
5x5	0.929	0.932	0.926	0.3%	-0.2%
8x8	0.978	0.982	0.980	0.4%	0.3%
10x10	1.000	1.003	0.998	0.2%	-0.2%
15x15	1.039	1.043	1.036	0.4%	-0.3%
20x20	1.063	1.066	1.062	0.3%	0.0%
30x30	1.087	1.089	1.102	0.2%	1.4%
40x40	1.095	1.099	1.093	0.3%	-0.2%
5x20	0.973	0.973	0.971	0.1%	-0.2%
20x5	0.971	0.971	0.972	0.7%	0.1%
			root mean squared:	0.57%	0.60%

Off-axis fields

Table 3A. Output factors for asymmetric fields, 6 MV. The last two columns show the difference between the measured and calculated output factors, for the two dose algorithms. The last row shows the average (root mean squared) difference over all fields.

field size (cm²)	offset X (cm)	offset Y (cm)	output factor measured	output factor CC	output factor MC	difference CC	difference MC
10x15	15	7.5	1.040	1.037	1.023	-0.3%	-1.6%
10x10	5	-5	1.018	1.010	1.011	-0.8%	-0.7%
10x10	5	0	1.015	1.016	1.017	0.1%	0.2%
10x10	-5	5	1.014	1.010	1.009	-0.4%	-0.5%
10x10	-5	0	1.011	1.016	1.010	0.5%	-0.1%
10x10	0	5	1.017	1.017	1.016	0.0%	0.0%
10x10	0	-5	1.021	1.017	1.015	-0.4%	-0.6%
					root mean squared:	0.4%	0.7%

Table 4A. Output factors for asymmetric fields, 6 MV FFF. The last two columns show the difference between the measured and calculated output factors, for the two dose algorithms. The last row shows the average (root mean squared) difference over all fields.

field size (cm^2)	offset X (cm)	offset Y (cm)	output factor measured	output factor CC	output factor MC	difference CC	difference MC
10x15	15	7.5	0.543	0.528	0.524	-2.8%	-3.6%
10x10	5	-5	0.801	0.797	0.798	-0.5%	-0.4%
10x10	5	0	0.878	0.875	0.880	-0.3%	0.2%
10x10	-5	5	0.803	0.797	0.800	-0.8%	-0.5%
10x10	-5	0	0.875	0.875	0.874	0.0%	-0.1%
10x10	0	5	0.880	0.876	0.880	-0.5%	-0.1%
10x10	0	-5	0.878	0.876	0.877	-0.2%	-0.1%
					root mean squared:	1.2%	1.4%

Geometrical test fields

Table 5A. Output factors for geometrical test fields, 6 MV. Monte Carlo calculations were performed at 0.1% uncertainty. The last two columns show the difference between the measured and calculated output factors, for the two dose algorithms. The last row shows the average (root mean squared) difference over all fields.

Veld	output factor measured	output factor CC	output factor MC	difference CC	difference MC
H-shaped field	0.874	0.894	0.887	2.3%	1.5%
Triangle	0.955	0.968	0.965	1.3%	1.0%
Dumbbell sym.	0.817	0.815	0.808	-0.2%	-1.1%
Dumbbell asym.	0.896	0.911	0.912	1.5%	1.7%
C-shaped field	0.862	0.887	0.882	2.8%	2.2%
			root mean squared:	1.8%	1.6%

Table 6A. Output factors for geometrical test fields, 6 MV FFF. Monte Carlo calculations were performed at 0.1% uncertainty. The last two columns show the difference between the measured and calculated output factors, for the two dose algorithms. The last row shows the average (root mean squared) difference over all fields.

Veld	output factor measured	output factor CC	output factor MC	difference CC	difference MC
H-shaped field	0.907	0.920	0.906	1.3%	-0.2%
Triangle	0.984	0.976	0.972	-0.8%	-1.3%
Dumbell sym.	0.843	0.852	0.855	1.1%	1.3%
Dumbbell asym.	0.938	0.937	0.935	-0.1%	-0.4%
C-shaped field	0.899	0.914	0.907	1.6%	0.8%
			root mean squared:	1.1%	0.9%

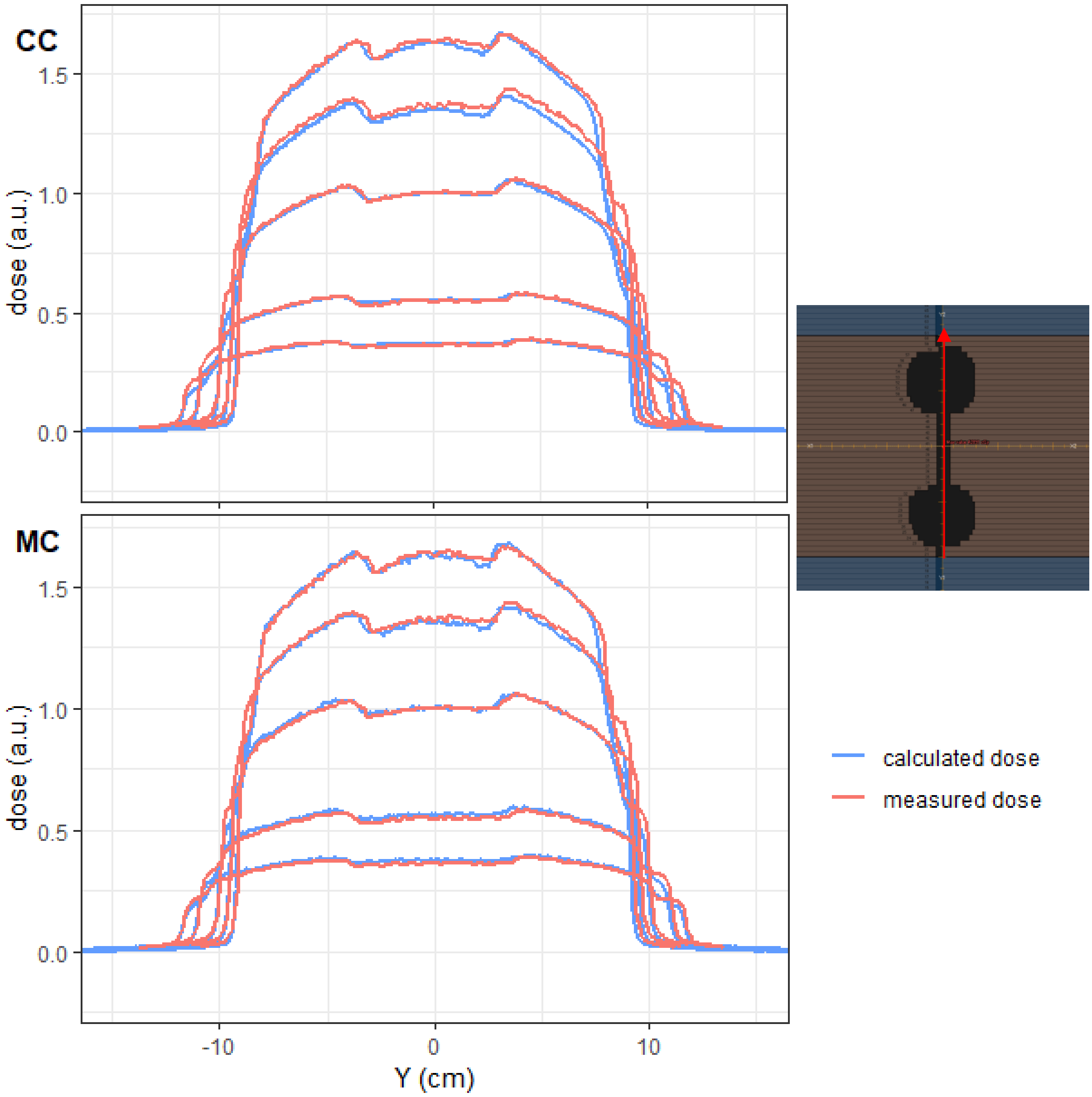

Figure 3A. Dose profiles at different depths for 6 MV FFF halter-shaped field (see image on the right for beams eye view of the leafs and Y-jaw positions), calculated with CC and MC.
